# Reduced NCK1 participates in unexplained recurrent miscarriage by regulating trophoblast functions and macrophage proliferation at maternal-fetal interface

**DOI:** 10.1590/1678-4685-GMB-2022-0297

**Published:** 2023-06-23

**Authors:** Chuanfeng Ding, Donghai Zhang, Shihua Bao, Xin Zhao, Yongsheng Yu, Qian Zhou

**Affiliations:** 1 Tongji University, School of Medicine, Shanghai First Maternity and Infant Hospital, Shanghai Institute of Maternal-Fetal Medicine and Gynecologic Oncology, Shanghai Key Laboratory of Maternal Fetal Medicine, Clinical and Translational Research Center, Shanghai, China.; 2 Tongji University, School of Medicine, Shanghai First Maternity and Infant Hospital, Shanghai Institute of Maternal-Fetal Medicine and Gynecologic Oncology, Shanghai Key Laboratory of Maternal Fetal Medicine, Department of Reproductive Immunology, Shanghai, China.; 3 University of Chinese Academy of Sciences, Chongqing School, Chongqing, China.; 4 Chinese Academy of Sciences, Chongqing Institute of Green and Intelligent Technology, Chongqing, China.

**Keywords:** NCK1, PD-L1, unexplained recurrent miscarriage, macrophage

## Abstract

Recurrent miscarriage (RM) seriously affects the physical and mental health of women of childbearing age, and 50% of the causes are unknown. Thus, it is valuable to investigate the causes of unexplained recurrent miscarriage (uRM). Similarities between tumor development and embryo implantation make us realize that tumor studies are informative for uRM. The non-catalytic region of tyrosine kinase adaptor protein 1 (NCK1) is highly expressed in some tumors, and can promote tumor growth, invasion and migration. In this present paper, we firstly explore the role of NCK1 in uRM. We find that the NCK1 and PD-L1 are greatly reduced in peripheral blood mononuclear cells (PBMC) and decidua from patients with uRM. Next, we construct NCK1-knockdown HTR-8/SVneo cells, and find that NCK1-knockdown HTR-8/SVneo cells exhibit reduced proliferation and migration ability. Then we demonstrate that the expression of PD-L1 protein is decreased when the NCK1 is knocked down. In co-culture experiments with THP-1 and differently treated HTR-8/SVneo cells, we observe significantly increased proliferation of THP-1 in NCK1-knockdown group. In conclusion, NCK1 may be involved in RM by regulating trophoblast proliferation, migration, and regulating PD-L1-mediated macrophage proliferation at the maternal-fetal interface. Moreover, NCK1 has the potential to be a new predictor and therapeutic target.

## Introduction

Recurrent miscarriage (RM) is defined as two or more spontaneous abortions before 20 weeks of gestation. RM is a multifactorial disease that affects a great number of couples. Miscarriage occurs in about 15% of normal couples (Alijotas-Reig and Garrido-Gimenez, 2013), and the incidence of RM in pregnant women is up to 2-4% (Khalife et al., 2019). Researchers have grappled with the causes of RM, and found that the main causes include chromosomal abnormalities, structural abnormalities, infections, immune dysfunction and thrombophilic disorders (Rai and Regan, 2006). However, approximately 50% of cases still cannot be explained by existing causes, and these cases are known as unexplained recurrent miscarriages (uRM) (Alijotas-Reig and Garrido-Gimenez, 2013). Therefore, it is worth the effort to reveal the causes of uRM.

Embryo implantation and placenta formation, which depend on the proliferation, migration and invasion of trophoblast cells, as well as immune homeostasis at the maternal-fetal interface, are essential to maintain pregnancy. Reduced trophoblast proliferation and migration were closely associated with RM (Ding et al., 2019). Macrophages are the second-largest immune cell population at the maternal-fetal interface, and macrophage-derived growth factors promote uterine artery remodeling (Shapouri-Moghaddam et al., 2018). Abnormal macrophage numbers and imbalance of M1/M2 subtype polarization are both potential causes of RM (Zhang J et al., 2022; Huang et al., 2021). The similarity of tumor cell-induced immune responses and the immune microenvironment at the maternal-fetal interface suggests potential similarities in pregnancy-related disease and tumor development (Mor et al., 2017). Thus, the exploration of uRM can be referred to the study of tumors.

The non-catalytic region of tyrosine kinase adaptor protein 1 (NCK1) contains three SH3 domains at the amino terminus and one SH2 domain at the carboxyl terminus (Paensuwan et al., 2016). Recent studies have found that NCK1 had the ability to positively regulate cell proliferation and migration (Dubrac et al., 2018; Liu et al., 2020). Moreover, NCK1 played a key role in enhancing TCR signal strength (Roy et al., 2010), and in the differentiation of CD4^+^ helper T cells (Lu et al., 2015), implying that NCK1 has immunomodulatory functions.

Programmed cell death 1 ligand 1 (PD-L1) is a transmembrane molecule, which is strongly associated with immune modulation via interaction with its receptor programmed cell death-1 (PD-1) (Beenen et al., 2022). Upregulated PD-L1 on tumor cells has the ability to inhibit the proliferation and infiltration of T cells (Hu et al., 2022). Moreover, PD-L1 can transmit a negative signal to tumor-associated macrophages to inhibit macrophage proliferation (Hartley et al., 2018). In addition, PD-L1 affected the proliferation, migration and invasion of tumor cells (Eichberger et al., 2020; Cao et al., 2022). Studies have shown the importance of PD-L1/PD-1 interaction in the maintenance of pregnancy, and PD-L1 expression was decreased in decidual immune cells from RM patients compared with normal early pregnancy (Meggyes et al., 2019).

In the current study, we firstly reported reduced NCK1 and PD-L1 in decidual tissue and peripheral blood mononuclear cells (PBMC) from uRM patients compared with normal control. Furthermore, we found that NCK1 may regulate trophoblast proliferation and migration, and PD-L1-mediated macrophage proliferation.

## Subjects and Methods

### Patients

Human samples from patients with uRM (n=20) and normal pregnant women (n=20) in the first trimester were collected from the operating room of the outpatient family planning department of Shanghai First Maternity and Infant Health Hospital from June 2020 to June 2021. The study was conducted in accordance with the Declaration of Helsinki, and approved by the Medical Ethical Committee of Shanghai First Maternity and Infant Hospital. Informed consent was obtained from all subjects involved in the study. Clinical characteristics of two group women were summarized in Table 1. The patients with a history of more than two consecutive pregnancy losses of unknown cause were include in uRM group. Exclusion criteria for the patients with uRM include fetal and parental chromosomal disorders, infectious diseases, genital tract malformation, autoimmune disorders, endocrinological dysfunctions and deficiencies in coagulation factors. The women with normal early intrauterine pregnancy were included in the control group. The women in the two groups had no history of exposure to sexually transmitted infection, no smoking or drinking habits, no exposure to radioactive substances or toxic and harmful chemicals during early pregnancy, and no chronic diseases.

**Table 1 -:** Clinical characteristics of normal pregnant women and patients with uRM.

Groups	Normal pregnancy (n = 20)	uRM (n = 20)	P value
Ages of pregnant women (years)	29.30± 3.00 (24-34)	30.60 ±3.01 (26-37)	>0.05
Length of pregnancies (days)	51.40 ±9.37 (41-86)	63.8±7.14 (56-83)	<0.01

### Tissue collection

Peripheral blood mononuclear cells (PBMC) and aborted tissue (villous tissue and decidual tissue) were collected from patients with uRM and normal pregnant women with active induced abortion in the first trimester. The informed consent was obtained from each patient before the study. Peripheral blood was collected from the outpatient biochemical laboratory, then were used for the extraction of PBMC. After induced abortion, most of the villous and decidual tissues were naturally separated. Trophoblast-rich villous tissues were identified among the aborted tissues, washed and collected, and the rest was decidual tissues. After residual villous tissues and blood clots on the surface of decidual tissues were removed, decidual tissues were collected. Then, the collected samples of villous and decidual tissues were stored in liquid nitrogen.

### Western blotting

Total protein was extracted using RIPA buffer (Sigma, USA) and protein concentration was determined using BCA protein kit (Thermo, USA). 5 µg of each protein was added to sodium dodecyl sulfate-polyacrylamide gel (SDS-PAGE) for electrophoresis. The proteins separated in the gel were transferred to a 0.22 µm diameter Immobilon-P membrane (Millipore, USA), and then the membrane was blocked in 5% skim milk (Beyotime, China) for 2 h at room temperature. Membranes were incubated with NCK1 (Abcam, USA), PD-L1 (Abcam, USA), GAPDH (Abways, China) antibodies overnight at 4 °C, and then added with Horseradish peroxidase-conjugated goat anti-rabbit/goat anti-mouse antibodies were incubated for 1 h at room temperature. Then ECL (Tanon, China) was used to detect the amount of protein.

### Real-time quantitative reverse transcription-polymerase chain reaction (RT-qPCR).

The cDNA of human PBMC samples was prepared using Takara reverse transcription kit, and mRNA expression was determined by real-time PCR machine (Life technologies, USA) using Real-time PCR kit (Yeasen, China). All data were normalized to Actin expression. The primer sequence is designed and synthesized according to the NCBI gene sequence, and the sequence is shown in Table 2.

**Table 2 -:** List of primers used for RT-qPCR.

Gene		Sequence (5’ to 3’)
*NCK1*	Forward	AATACTGGGCAAGTGTTGCAT
	Reverse	TTTCCACCACTCTGGGTCATT
*PD-L1*	Forward	ATGTGACCAGCACACTGAGA
	Reverse	TCAGTGCTACACCAAGGCAT
*ACTIN*	Forward	ACTCGTCATACTCCTGCT
	Reverse	GAAACTACCTTCAACTCC

### Fluorescence immunohistochemistry

The decidual tissues were embedded in OCT (Sakura, USA), wrapped in tin foil, frozen in liquid nitrogen, and sliced using fast freezing microtome. Sections were fixed with 4% paraformaldehyde, washed and blocked with 5% BSA, and sequentially incubated with a primary antibody for 2 h and a secondary antibody for 1 h. After adding DAPI (Abcam, USA), they were photographed using a fluorescence microscope (Leica, TCS SP8 CARS).

### Cell culture

Human trophoblast cell line HTR-8/SVneo cells and human monocyte cell line THP-1 cells were obtained from the American Type Culture Collection (ATCC). The HTR-8/SVneo and THP-1 cells were cultured in RPMI1640 medium (Gibco, USA) containing 10% fetal bovine serum (Gibco, USA) and 1% penicillin/streptomycin at 37 °C, 5% CO2.

### RNA interference

Small RNA interference (siRNA) was used in this study. siRNA for the NCK1 gene was purchased from Hanbio Biotechnology (China) and transfected into HTR-8/SVneo (HTR-8-siNCK1) cells using Lipo 3000 (Invitrogen, USA) according to the manufacturer’s instructions. The siRNA target sequence is (5^’^-3^’^): Sense strand: GCAUCCCUGCUGAUGAUATT and Antisense strand: UAUCAUCAGCAGGAGAUGCTT. The HTR-8/SVneo cells transfected with a scrambled siRNA were considered as negative control (HTR-8-NEG). 

### Cell proliferation assay

HTR-8/SVneo cells under different interventions were plated at 2000 cells per well in a 96-well plate, and cultured in a 37 °C cell incubator for 24 h, 48 h, and 72 h. Cell proliferation was measured using a Cell Counting Kit-8 (CCK-8; Beyotime, China) according to the manufacturer’s instructions. The absorbance of each sample was measured at 450 nm wavelength in a microplate reader.

### Co-culture of HTR-8/SVneo and THP-1 cells

5×10^5^ HTR-8/SVneo cells under different interventions were plated in 24-well plates, and 5×10^5^ THP-1 cells was added in the 24-well plates the next day. After co-culture at 37 °C for 24 h, 48 h, and 72 h, the cell suspension containing THP-1 cells was aspirated and spread into a 96-well plate, and the absorbance at 450 nm wavelength was detected after Cell Counting Kit-8 (CCK-8; Beyotime, China) was added.

### Cell migration assay

Transwell 24-well plates (Corning, USA) were used for the cell migration assay. 200 μl of serum-free cell culture medium containing 1×10^5^ cells were added to each upper chamber of the Transwell plate, and 500 μl 10% FBS medium was added to each well of the lower chamber of the Transwell plate. The cells were incubated for 24 h in a cell incubator. The bottom of the upper chamber of the Transwell plate was fixed with 4% paraformaldehyde, stained with crystal violet, and no migrated cells were removed with a cotton swab, then the migrated cells were photographed under an inverted microscope.

### Statistical analysis

All data analyses were performed, and graphs were drawn using GraphPad Prism 8.0 software. Results were displayed as mean ± SD. The significant difference between groups was calculated by Student’s t-test; *P < 0.05* was considered to be statistically significant.

## Results

**Figure 1 -:**
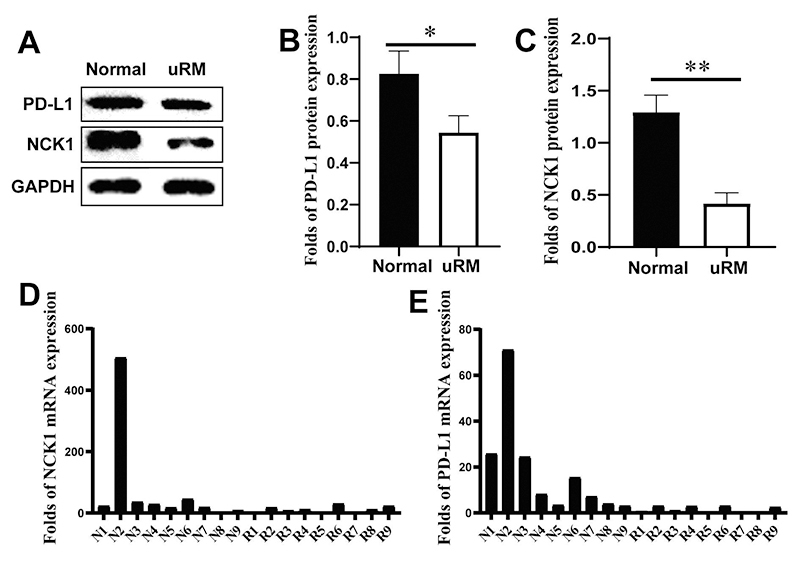
Protein and mRNA expressions of NCK1 and PD-L1 in PBMC of normal pregnant women and patients with uRM. (A-C) Expression of PD-L1 and NCK1 proteins in PBMC of normal pregnant women and patients with uRM detected by Western blotting. For data analysis, the Western blotting was repeated three times. (D-E) mRNA expression of *NCK1* and *PD-L1* in PBMC of normal pregnant women and uRM patients (N1-N9 were normal pregnant women; R1-R9 were patients with uRM) detected by RT-qPCR. (*p<0.05, **p<0.01).

**Figure 2 -:**
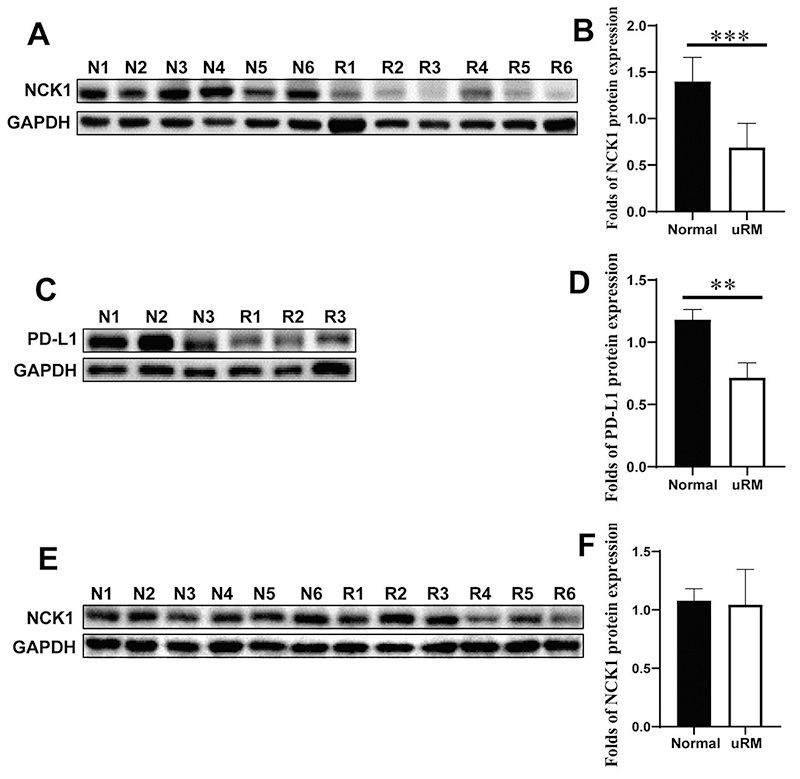
Expression of NCK1 and PD-L1 proteins in decidual and villous tissues of early normal pregnant women and patients with uRM. Expression of NCK1 (A, B) and PD-L1 (C, D) proteins in decidual tissues of early normal pregnant women and patients with uRM (N1-N6 were normal pregnant women; R1-R6 were patients with uRM) were detected by Western blotting. Expression of NCK1 protein (E, F) in villous tissues of normal pregnant women and patients with uRM were detected by Western blotting (N1-N6 were normal pregnant women; R1-R6 were patients with uRM). (**p<0.01, ***p<0.001).

**Figure 3 -:**
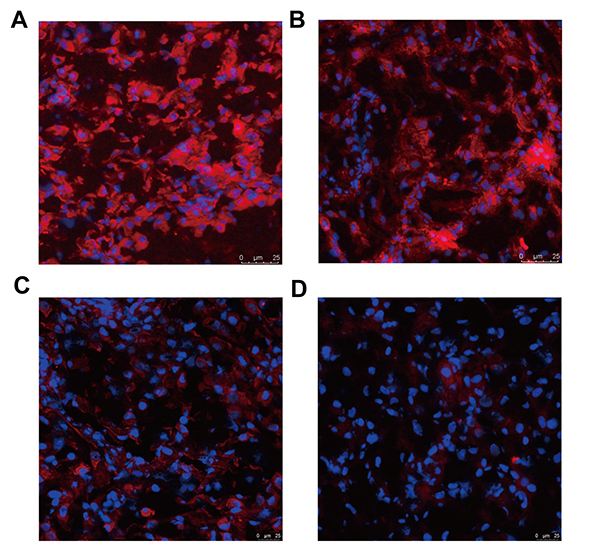
Fluorescence immunohistochemistry in decidual tissues. Fluorescence immunohistochemistry showed the expression of NCK1 (A) and PD-L1 (B) proteins in decidual tissues of early normal pregnant women. Immunohistochemistry showed the expression of NCK1 (C) and PD-L1 (D) protein in the decidual tissue of patients with uRM. Purple is the nuclear staining and red is the target protein staining.

We collected villous and decidual tissues from normal pregnant women and patients with uRM, and found that there was no statistical difference in age between the two groups, but the length of pregnancy was significantly longer in patients with uRM than in normal pregnant women. Table 1 shows the ages of pregnant women (years) and the length of pregnancies (days). The expression of NCK1 and PD-L1 proteins (Figure 1A-C) in PBMC of early normal pregnant women was greatly higher than those of patients with uRM. In addition, the expression of *NCK1* and *PD-L1* mRNA (Figure 1D, E) in PBMC of early normal pregnant women was also greatly higher than those of patients with uRM. Moreover, the expression of NCK1 and PD-L1 proteins in decidual tissues of early normal pregnant women was significantly higher than those of patients with uRM (Figure 2A-D). However, the expression of NCK1 protein in villous tissues of early normal pregnant women was not significantly different from those of patients with uRM (Figure 2E,F). In addition, fluorescence immunohistochemistry showed that the expression of NCK1 and PD-L1 proteins in the decidual tissues of early normal pregnant women was higher than those of patients with uRM (Figure 3A-D).

**Figure 4 -:**
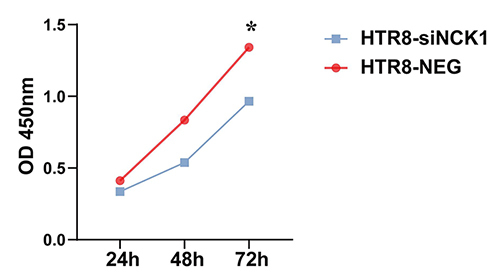
Knockdown of NCK1 reduced HTR-8/SVneo proliferation. OD value of HTR-8/SVneo cells with NCK1 knockdown at 450 nm wavelength. The CCK8 assay was repeated three times. (*p<0.05).

siRNA was successfully transfected into HTR-8/SVneo cells and we detected HTR-8/SVneo proliferation via CCK8 assay. The results showed that the HTR-8/SVneo proliferation was significantly decreased when NCK1 was knocked down (Figure 4).

**Figure 5 -:**
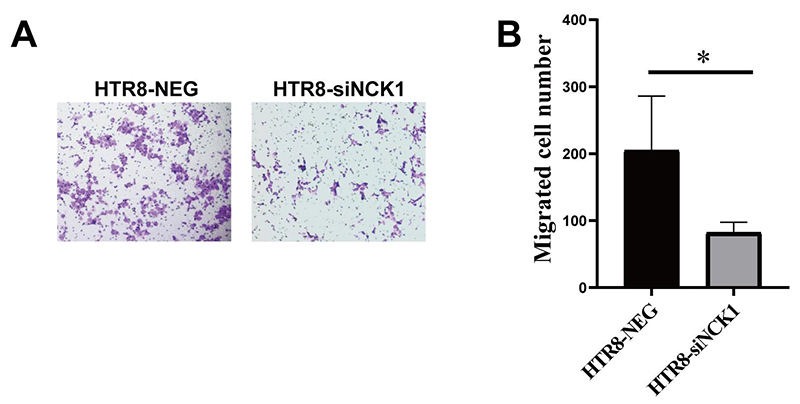
Knockdown of NCK1 reduced HTR-8/SVneo migration. (A) The migration picture of HTR-8-NEG group and HTR-8-siNCK1 group. The data analysis of (A) was presented in (B). Five microscopic fields were taken from each well for counting. (*p<0.05).

We explored the effect of NCK1 on the migration of trophoblast cells via Transwell migration assay. The HTR-8/SVneo cells under different interventions were cultured in the Transwell upper chamber for 24 hours, and the changes of cell migration ability were observed under a microscope (Figure 5A). We found that the number of migrated cells was significantly decreased in HTR-8-siNCK1 group than HTR-8-NEG group (Figure 5A,B).

**Figure 6 -:**
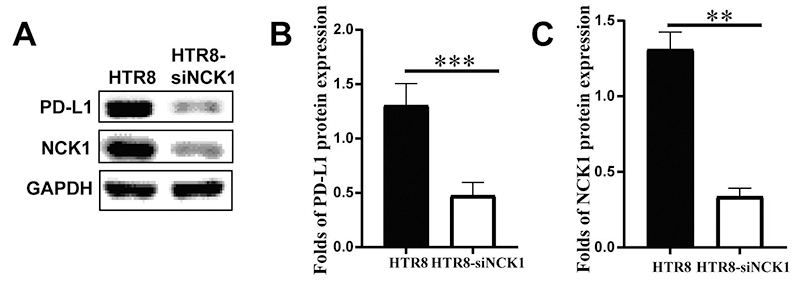
Expression of NCK1 and PD-L1 proteins in HTR-8/SVneo cells with NCK1 knockdown. (A) Expression of NCK1 and PD-L1 proteins was detected by Western blotting. Quantification of PD-L1 expression was presented in (B). Quantification of NCK1 expression was presented in (C). For data analysis, the Western blotting was repeated three times. (**p<0.01, ***p<0.001).

The expression of NCK1 and PD-L1 proteins in HTR-8-siNCK1 group and HTR-8-NEG group was detected via Western blotting. The results showed that compared with the HTR-8-NEG group, the expression of NCK1 and PD-L1 proteins in HTR-8-siNCK1 group was greatly decreased (Figure 6A-C).

**Figure 7 -:**
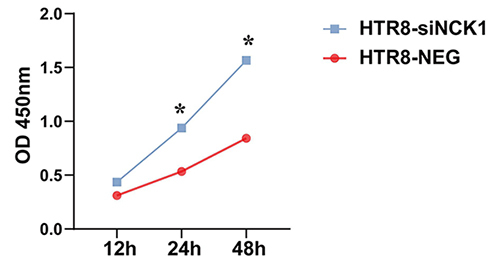
Co-culture of HTR-8-siNCK1 and THP-1 enhanced THP-1 proliferation. OD values of THP-1 cells co-cultured with HTR-8-siNCK1 cells and HTR-8-NEG cells at 450 nm wavelength. The CCK8 assay was repeated three times. (*p<0.05).

After co-culture with the THP-1 and HTR-8/SVneo cells under different interventions, we detected the proliferation of THP-1 via CCK8 assay. The results showed that the proliferation of THP-1 co-cultured with HTR-8-siNCK1 group was significantly increased than co-cultured with HTR-8-NEG group (Figure 7).

## Discussion

RM seriously affects the physical and mental health of pregnant women. Since the causes of miscarriage are poorly understood, no effective treatment has emerged. Thus, it is of great significance to explore the pathological mechanism to prevent and treat uRM. In recent years, studies have confirmed that NCK1 is highly expressed in some tumors, and is associated with enhanced tumor proliferation, migration and invasion (Liu et al., 2020; He et al., 2022). The similarity between embryo implantation and tumor development is widely recognized (Mor et al., 2017). Thus, the study of NCK1 in tumors may expand our knowledge of uRM.

We included 20 normal pregnant women and 20 patients with uRM in this study. Abortions in the control group were performed earlier than occurred in the group of recurrent abortions. This difference may be due to the delayed artificial abortion in patients with uRM who try to maintain their pregnancy via continuous treatment. Although the samples from the elective abortion group were obtained a little earlier (approximately 10 days) than those of uRM, we believe this difference did not interfere with the results obtained, considering both are included in the first trimester of pregnancy. Our results showed that protein and mRNA expressions of NCK1 and PD-L1 were significantly reduced in PBMC from uRM than from control, implying reduced NCK1 in PBMC may become a new predictor for uRM. Moreover, in immunofluorescence reactions, the tissue level of both PD-L1 and NCK1 was reduced in the decidua, which corroborated the data obtained for expression of NCK1 and PD-L1 proteins in the decidua. Between villous tissues from patients with uRM and control, we did not find differences in the expression of NCK1 protein. Based on these results, we proposed a hypothesis that NCK1 and PD-L1 might be intrinsically linked. 

HTR-8/SVneo is a cell line commonly used to explore trophoblast function (Zhang R et al., 2022; Zhang D *et al.*, 2023), and in this study we found that HTR-8/SVneo expressed NCK1 via Western blotting. Thus, to further explore the possible mechanism of NCK1 in uRM, we selected the trophoblast cell line HTR-8/SVneo. We used siRNA to knock down the expression of NCK1 in HTR-8/SVneo. Then we examined the effect of NCK1 on trophoblast proliferation using CCK8 assay, and found that NCK1 promoted trophoblast proliferation. Moreover, we examined the effect of NCK1 on trophoblast migration using Transwell migration assay and found that NCK1 promoted trophoblast migration. In HTR-8/SVneo, when NCK1 was knocked down, the expression of PD-L1 protein was significantly reduced compared with negative control. This result suggested a regulatory interaction between NCK1 and PD-L1. However, the exact regulatory mechanism has not been reported. Next, in first-trimester pregnancy, some cytotrophoblasts migrated towards the decidua, and invasive cytotrophoblasts interacted with immune cells in the decidua (Zhang D *et al.*, 2022). Moreover, previous studies have found that macrophages were the second most abundant immune cell in the decidua, and that dysregulated interaction between macrophages and trophoblasts might be a cause of RM (Zhang D *et al.*, 2022). Thus, we explored the effect of NCK1 on the proliferation of macrophages at the maternal-fetal interface using co-cultures of THP-1 and differently treated HTR-8/SVneo. We found that the proliferation of macrophages was significantly increased in the HTR-8-siNCK1 group. Previous studies revealed that PD-L1 has the ability to regulate macrophage proliferation (Hartley et al., 2018; Zhang Y *et al.*, 2019). Thus, these results suggested that NCK1 may regulate PD-L1-mediated macrophage proliferation. Previous studies have reported that RM was closely associated with immune dysfunction, such as imbalance of macrophage polarization and dysfunction of macrophage cytokine secretion (Zhao et al., 2022). Thus, enhanced proliferation of macrophages may be detrimental to pregnancy maintenance by causing immune dysfunction at the maternal-fetal interface. 

**Figure 8 -:**
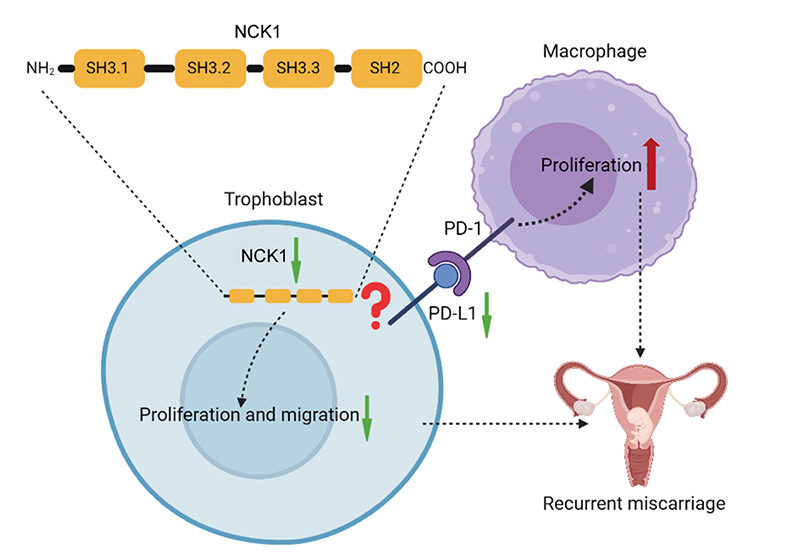
Possible mechanisms of NCK1 involvement in recurrent miscarriage. Reduced NCK1 on trophoblast cells may be a cause of recurrent miscarriage. On the one hand, reduced NCK1 inhibits the migration and proliferation of trophoblast cells, on the other hand, reduced NCK1 promotes macrophage proliferation by attenuating PD-L1 expression, ultimately leading to an immune imbalance in the maternal-fetal interface.

Overall, in this study, we found that reduced NCK1 may be involved in RM by inhibiting trophoblast proliferation and migration, as well as enhancing PD-L1-mediated macrophage proliferation at the maternal-fetal interface (Figure 8). However, the exact regulatory mechanism between NCK1 and PD-L1 is still unclear. Moreover, NCK1 has the potential to be a new predictor and therapeutic target.
